# Tail risk connectedness in clean energy and oil financial market

**DOI:** 10.1007/s10479-022-04745-w

**Published:** 2022-05-15

**Authors:** Matteo Foglia, Eliana Angelini, Toan Luu Duc Huynh

**Affiliations:** 1 Department of Economics, Management and Business Law, “Aldo Moro” University of Bari, Bari, Italy; 2 Department of Economics, “G.d’Annunzio” University of Pescara, Viale Pindaro 42, 65127 Pescara, PE Italy; 3 Southampton Business School, University of Southampton, Southampton, SO17 1BJ UK

**Keywords:** Clean energy firms, Oil firms, Tail risk spillover, Interconnectedness, Spillover network, C58, G10, Q42

## Abstract

This research investigates the connectedness and the tail risk spillover between clean energy and oil firms, from January 2011 to October 2021. To this, we use the Tail-Event driven NETworks (TENET) risk model. This approach allows for a measurement of the dynamics of tail-risk spillover for each sector and firm. Hence, we can provide a detailed picture of the existing extreme relationships within these markets. We find that the total connection between the markets varies during the period analysed, showing how the uncertainty in oil price plays a critical role in the risk dynamics for oil companies. Also, we find that relationships between energy firms tend to be intrasectoral; that is, each sector receives (emits) risk from (to) itself. These results can have important practical implications for risk management and policymakers.

## Introduction

“...the European Commission’s new sustainable finance strategy, the Network for Greening the Financial System scenarios should be considered a starting point for testing the impact of Europe-specific transition plans...”

Frank Elderson

Member of the Executive Board of the ECB

Vice-Chair of the Supervisory Board of the ECB

Promoting sustainable development and the fight against climate change have become integral aspects of energy planning and policymaking. It is not only the puzzle of environmental economics but also the concern of the financial world, where investors could (or could not) direct their capital flows to either ‘green’ or ‘grey’ energy.^1^ More noticeably, the demand for global energy requires an optimal way to allocate energy in a portfolio to achieve carbon-neutral strategies with less harm to flows in sustainable investments. Inspired by the speech of Frank Elderson, this paper constructs the scenarios of the network for the green and financial system when exploring its nexus with finance energy. Accordingly, because of the flourishing of investments in sustainable energy, investors currently have more choices apart from traditional commodities such as crude oil. Indeed, in recent years, the relationship between clean energy stock price and oil price has been a popular topic in the field of energy finance. By their nature, these two stock markets are intrinsically linked. As well highlighted by Reboredo (2015), the performance of renewable energy firms is influenced by the dynamics of the oil market, making it more or less profitable to replace exhaustible energy resources with sustainable energy resources (Kumar et al., 2012). Accordingly, building a risk model to detect the combination of sustainable investments and financial instruments is really promising. Therefore, this study is motivated to contribute empirical evidence with a cutting-edge model for both clean energy and oil firms.

Concomitantly, this paper is motivated by uncertainties, which might cause tail interdependencies among different financial assets. Especially, the world has faced unprecedented events such as the COVID-19 pandemic (Huynh et al., 2021; Managi et al., 2022; Chai et al., 2022), Eurozone shocks (Foglia et al., 2022), and even the global network (Nguyen & Lambe, 2021). Hence, a detailed understanding of how the energy and financial markets are connected would identify the market structure during normal turbulence. Let us imagine that investors invest in clean energy, but they might miscalculate the potential risks. Therefore, investors who favour holding portfolios with energy assets and financial instruments should have been concerned about intertail risk to avoid extreme losses. *Why tail risk?* The current literature represents that the dynamics and comovements of asset returns would be associated with tail risk (Ang & Chen, 2002; Madaleno & Pinho, 2012; Nguyen & Lambe, 2021). In addition, green financial instruments are not as popular as other investments because of lower attention (Pham & Huynh, 2020). Thus, we emphasise the need to model tail risk for the network of both energy instruments and stock returns

Earlier studies have explored the spillover effects between oil price and clean energy stock price at an aggregate level using clean energy indices (e.g. Henriques & Sadorsky, 2008; Managi & Okimoto, 2013; Reboredo, 2015; Bondia et al., 2016; Ahmad, 2017; Ferrer et al., 2018; Saeed et al., 2021). However, analysis at an aggregate level cannot capture all links within the clean energy and oil sector, overlooking the heterogeneity within the relationship. In this article, we take the first step to document this connection by exploring the tail-risk interaction between 32 firms in the clean energy and oil sectors. Understanding these links is crucial in the current context of the sustainable development of energy sources. For this purpose, the paper seeks to fill this gap by contributing to the existing literature in several ways.

First, the research enhances the literature that analyses the risk spillover effect between oil and clean energy at the firm level. Indeed, to the best of our knowledge, only three works have been conducted at the firm level. For example, Restrepo et al. (2018), studied the volatility spillover between stock returns of the 20 biggest oil firms, while Antonakakis et al. (2018) examined optimal weights and hedge ratios among oil companies. More recently, Foglia and Angelini (2020), using Diebold and Yılmaz (2014)’ model, analysed the volatility spillover between clean energy firms and oil prices. In our research, using the the Tail-Event driven NETwork (TENET, Härdle et al., 2016) risk model, we are able to offer fresh information on the degree of interconnection (spillover effect) between markets, providing a detailed picture of the relationship. Although many works have analysed this relationship using several different methodologies such as the VAR and VECM models (Henriques & Sadorsky, 2008; Kumar et al., 2012; Managi & Okimoto, 2013; Bondia et al., 2016; Chai et al., 2022); wavelets (Reboredo et al., 2017); copulas (Reboredo & Ugolini, 2018); GARCH and variants (Sadorsky, 2012a; Lv et al., 2021, 2012, 2014) and framework and variants (Ahmad, 2017; Ferrer et al., 2018; Pham, 2019; Nasreen et al., 2020; Foglia & Angelini, 2020), none of these methods have been able to capture extreme spillover effects from a network perspective at the firm level. Our paper is close to a recent work of Saeed et al. (2021) who, by extending the Diebold and Yılmaz (2012, 2014)’ model in a quantile perspective, managed to model spillover effects between dirty and clean markets. However, we differ from their paper because our research is at the firm level. Second, by using TENET, we are able to estimate a nonlinear relationship between assets in contrast to the quantile-VAR (QVAR) method. Third, by mixing balance sheet and stock market data, our measure captures both market sentiment and the economic and financial strength of various industries. Fourth, TENET is a weighted and directed network, while the network of models Diebold and Yılmaz (2012, 2014), such as TVP-VAR and QVAR, are not weighted and directed. Therefore, TENET includes more information and has more power to capture the extreme risk or tail event than the mean spillover network (such as Ferrer et al., 2018). In fact, our approach, in contrast to the mean estimation regression (such as DCC-GARCH, TVP-VAR, Diebold and Yılmaz (2012) models), does not impose any assumptions on the distributions and allows us to study the extreme spillover effects on the network system. Finally, we are able to investigate the contribution of each green and brown firm by considering their tail interconnects with other energy companies (i.e., we consider all the systems) in contrast to Lv et al. (2021), who, by BEKK-GARCH-M could analyse only the bidirectional relationship.

Our main results can be summarised as follows. From January 2011 to October 2021, for 24 firms in the clean energy sector (wind, solar and energy efficiency) and 8 companies belonging to the oil industry, we find that each company receives (emits) more tail risk from (to) its industry, showing a sector cluster. Moreover, the analysis reveals a decoupling between the clean energy sector from the oil market (poor tail-risk spillover). This detailed information helps clarify the policy incentive mechanism. For example, the few links between oil and clean energy companies mean that the renewable energy sector does not need specific policies to protect against the impact of oil market fluctuations (Ferrer et al., 2018). Second, we find that both total and cross-sector connections reach a peak when the energy market is uncertain (e.g., a drop in oil price). This knowledge allows regulators to use these indices as early-warning indicators for energy system distress. Indeed, by estimating the total connection and identifying the companies that transmit and receive tail risk, we help managers to mitigate the risks arising from the energy financial markets. Third, we quantify the risk contribution of each industry (firm) to the whole energy system. For instance, we find that firms belonging to the solar sector are always net transmitters of tail risk, unlike oil companies, which are net risk receivers. This information can be useful to investors in their portfolio strategies, investing in the oil or renewable energy market depending on risk level.

The remainder of the paper is organised as follows. Section 2 provides the literature review. We present the empirical model in Sect. 3. Section 4 gives an overview of the data. In Sect. 5, we discuss our empirical results, and in Sect. 6, we provide some concluding remarks.

## Literature review

In recent years, the growth of the clean energy sector has created an emerging body of research focusing on the relationship between oil prices and clean energy markets. The literature can be classified into three main categories according to the aims and econometric models applied. The first category studies the stock price relationship between these markets. A pioneering work is the study of Henriques and Sadorsky (2008) who, using a vector autoregressive model (VAR), found a linear Granger causality from crude oil to clean energy stock prices. However, the authors show that the impact of the technology sector on renewable energy is more significant than that of oil prices. Kumar et al. (2012) added variables to Henriques and Sadorsky (2008)’ analysis, such as coal price and interest rate. Their results indicated that past movements in oil prices, high-tech companies’ stock prices and interest rates affect the price dynamics of clean energy. They also showed the close link between clean energy and the technology sector, as well as the research of Managi and Okimoto (2013). The authors, using a Markov-switching vector autoregressive model, were able to capture structural changes in the relationship between oil stock prices and clean energy. They found that before 2007 (structural break), oil prices did not affect clean energy stock prices, while there was a positive relationship after the break. The study of Inchauspe et al. (2015) confirmed these results. Using a state-space model, they revealed a high level of correlation between the MSCI World Index, technology stock returns and clean energy. Meanwhile, they found a weak influence on oil price. Bondia et al. (2016) applied a threshold cointegration analysis to verify the causality between clean and oil stock price. The Granger test suggested a significant relationship only in the short run. Nevertheless, the link was not significant in the long run. In contrast, Reboredo et al. (2017), using continuous and discrete wavelets, found that relationship dependence between oil and renewable energy returns is weak in the short run, but it increases over the long term. Dimitriadis and Katrakilidis (2020) computed a cointegration analysis to study the dynamic relationship among ethanol, crude oil and corn market prices. Their findings provided a significant positive long-run causal relationship.

The second category focuses on the study of risk spillover. For example, Sadorsky (2012a) employed a multivariate GARCH model to study volatility spillovers between oil prices and clean energy stock prices. Their results confirmed that clean energy stock prices are strongly correlated with technology stock prices. Reboredo (2015) applied a copula model and CoVaR measure to study the effect of oil price on renewable energy stock return. They found that oil price dynamics contribute around 30% to the downside and upside risk (CoVaR) of renewable energy firms. The same evidence was provided in Reboredo and Ugolini (2018). Applying a multivariate wine-copula dependence, the authors revealed that energy price movements play an essential role in the dynamics of the renewable energy financial market, especially when energy prices are subject to downward or upward fluctuations. Lin and Li (2015), using a VEC-MGRACH model, studied the spillover effect between natural gas and oil prices. The authors showed that European and Japanese gas prices are cointegrated with Brent oil prices, while the natural gas price in the United States is decoupled with crude oil price in the long term. Moreover, they found a bidirectional volatility spillover effect between the oil market and natural gas market both in the United States and in Europe. On the contrary, they found that the volatility in Japan is independent of the natural gas and oil markets. Dutta (2017) documented the significant impact of the crude oil volatility index (OVX) on clean energy stock returns. Further, Uddin et al. (2019), using cross-quantilogram correlation, found a positive dependence between renewable energy stock return and several asset prices. In particular, the authors showed that the returns of renewable energy influence oil when both are at their lowest quantile. Meanwhile, an increase in oil leads to growth in renewable energy performance. More recently, Lv et al. (2021), by using a K-GARCH-M model, studied the spillover effect between oil price and Chinese clean energy subsectors (such as hydropower, solar energy, nuclear power, wind energy, new energy and new-energy vehicles). Their analysis was conducted at sectoral index levels. The authors found a strong relationship between petroleum prices and the new-energy-vehicle sector. In addition, their results showed that the relationship between the clean sector and oil price is bidirectional.

Moreover, to analyse the spillover effects and network characteristics of these markets, many papers used Diebold and Yılmaz (2012, 2014) models. For example, Ahmad (2017) found that technology and clean energy stocks are net emitters of returns, while crude oil is the net receiver. The author highlighted the critical role of technology stocks in the volatility fallout from renewable energy stocks and crude oil prices. In addition, the results showed the limited interdependence between oil prices on clean energy and technology indices. Ferrer et al. (2018) documented the same conclusions. Looking at the dynamics between U.S. alternative energy companies’ stock prices, crude oil prices and other financial variables (high technology and conventional energy stock prices, 10-year U.S. Treasury bond yields, U.S. default spread and volatility in U.S. stock and Treasury markets), the authors found that crude oil prices are not determinants of movements in clean energy companies’ stock prices. Focusing on four investment asset classes (stocks, currency, U.S. Treasury bonds and oil) and uncertainty measures, Lundgren et al. (2018) found that the European stock market depends on renewable energy prices. Their results illustrated how investments in the clean sector are riskier than in the nonrenewable sector. Xia et al. (2019), using Diebold and Yılmaz (2012) framework, analysed the extreme influence of energy price changes on renewable energy stock returns. Their findings revealed that, under extreme risk situations, the renewable energy market is the net risk contributor to fossil energy performance. Nasreen et al. (2020), to study volatility spillover among oil prices, clean energy and technology stock price, used three econometric techniques: (1) the multivariate GARCH model, (2) wavelet analysis and the (3) Diebold and Yılmaz (2012) model. Their findings showed (i) high persistence of volatility in future markets and (ii) a weak interdependence between clean energy and oil price. Of interest is the work of Pham (2019), who analysed the relationship between oil prices and clean energy stocks at the disaggregated level. In particular, the author used a wide range of stock indices to represent the clean energy sector, showing how the relationship between oil prices and clean energy prices is heterogeneous according to subsector indices. For example, biofuels and energy management stocks have the highest link to oil prices, while wind, geothermal and fuel cell stocks have the lowest. Saeed et al. (2021) used an innovative quantile VAR model to capture the extreme risk spillover between clean energy stocks, green bonds, crude oil and energy ETF. Their results showed an asymmetric effect across levels of connectedness. In particular, the authors found strong left and right tails relative to the median of the distribution. This result documents the importance of using quantile-based measures over mean-based measures (Saeed et al., 2021; Tiwari et al., 2021).

Moving toward firm-level analyses, Restrepo et al. (2018), analysed the volatility spillover between the stock returns of the 20 biggest oil firms. Using Diebold and Yılmaz (2012, 2014)’ framework, the paper highlighted how financial turmoil has a strong impact on the oil market. Also, the authors showed how oil firms are highly connected, i.e., a source of systemic risk. Antonakakis et al. (2018) examined optimal weights and hedge ratios among oil companies. The authors highlighted the key role played by oil firms in the spread of risk on the dynamic of WTI crude oil. The results evidenced how WTI crude oil is the net receiver of spillover shocks. More recently, Foglia and Angelini (2020), analysed the volatility spillover between clean energy firms and oil price. Their results highlighted how the spillover connectedness between the oil and clean energy sector reach their highest values in turbulent times. Studying heterogeneity at the corporate level is essential to understand, for example, the impact of oil shock on the clean and non-clean energy stock market. This information is relevant both to policymakers’ policies and to investors in terms of risk management. For example, traders should give importance to clean companies that act as net transmitters of volatility because they can influence the risk of other companies. Also, considering risk at the firm level allows us to understand the intrasectoral transmission of risk. If we look at financial firms, for example, they are classified in terms of global systemically important financial institutions. Therefore, if we were to consider risk at an aggregate level (financial sector), policymakers would not be able to make ad hoc interventions to avert systemically important financial risk. The same could apply to the world of energy companies. Classifying companies according to their contribution to risk would allow policymakers to make timely and targeted interventions. This could prevent the transmission of risk within other related and unrelated sectors. Therefore, the study could help create a kind of global systemically important energy firm.

Hence, in this paper, we extend their analysis using a new methodology that considers tail risk. In fact, because of the TENET model, we can consider a high dimension contest, the nonlinearity between pairs of series, and a weighted and direct network. Therefore, TENET is useful to investigate the contribution of each green and brown firm by considering its tail interconnects with other energy companies and to analyse the spillover effect. Further, as recommended by Saeed et al. (2021), using quantile-based measures of connectedness is essential to study the transmission of risk between these markets. Applying mean-based measures of connectedness in energy markets could lead to erroneous conclusions and mask important findings.

## Empirical methodology

### The TENET design

The TENET model allows for an analysis of tail-risk spillover between firms. Given the rapid growth of green finance and growing concerns about climate change, it is essential to capture the feedback effect of clean markets on the oil market (and vice versa) in terms of risk transmission. The model is an extension of the CoVaR measure proposed by Adrian and Brunnermeier (2016). The TENET framework has several interesting features. First, contrary to the CoVaR, which allows us to capture only the risk of “renewable energy stocks provided that oil markets experience extreme price fluctuations” (Reboredo, 2015), TENET considers the interaction of all systems (firms). Neglecting these effects may lead to an incorrect estimation of risk spillover. Second, using single-index quantile regression allows for an estimation of a nonlinear regression between firms, which is particularly important during financial turbulences (e.g., drop oil). Third, TENET provides several measures of connectedness to capture the tail-risk contributions for each company.

TENET consists of various steps. The first is to estimate the VaR and CoVaR for each firm by quantile (tail event) regression:

1 $$\begin{aligned}&X_{i,t}=\alpha _i + \gamma _i M_{t-1}+\varepsilon _{i,t}, \end{aligned}$$

2 $$\begin{aligned} \&X_{j,t}=\alpha _{j|i} + \gamma _{j|i} M_{t-1}+\beta _{j|i}X_{i,t} + \varepsilon _{j|i,t}. \end{aligned}$$

where $$X_{i,t}$$ and $$ X_{j,t}$$ are the log returns of firm *i* (*j*) at time *t*. $$M_{t-1}$$ is a vector of macro state variables. $$\gamma _i$$, $$\gamma _{j|i}$$ and $$\beta _{j|i}$$ are the slope coefficients. $$\beta _{j|i}$$ captures the conditional tail event risk, i.e, the sensitivity of firm *j* to changes in tail event log return of firm *i*. The $$VaR_{i,t,\tau }$$ and $$CoVaR_{j|i,t,\tau }$$ of firm *i* and *j* are computed by plugging the predictive values from the above estimates,

3 $$\begin{aligned}&\widehat{VaR}_{i,t,\tau }=\hat{\alpha _i} + \hat{\gamma _i} M_{t-1} \end{aligned}$$

4 $$\begin{aligned}&\widehat{CoVaR}_{j|i,t,\tau }=\hat{\alpha }_{j|i} + \hat{\gamma }_{j|i} M_{t-1}+\hat{\beta }_{j|i} \widehat{VaR}_{i,t,\tau } \end{aligned}$$

The second step concerns the use of single index quantile regression (SIM) to compute the interdependence network. Following Härdle et al. (2016), we define the directional spillover as follows:

5 $$\begin{aligned}&X_{j,t}=g (\beta _{j|R_j}^\top , R_{j,t}) +\varepsilon _{j,t}, \end{aligned}$$

6 $$\begin{aligned}&\widehat{CoVaR}^{TENET}_{j|\tilde{R}_{j},t,\tau } \equiv \hat{g} (\hat{\beta }_{j|\tilde{R}_j}^\top \tilde{R}_{j,t}), \end{aligned}$$

7 $$\begin{aligned}&\hat{D}_{j|\tilde{R}_j} \equiv \frac{\delta \hat{g} (\hat{\beta }_{j|\tilde{R}_j}^\top R_{j,t}) }{\delta R_{j,t}} |_{R_{j,t}=\tilde{R}_{j,t}} = \hat{g}' (\hat{\beta }_{j|\tilde{R}_j}^\top \tilde{R}_{j,t}) \hat{\beta }_{j|\tilde{R_j}}. \end{aligned}$$

where $R_{j,t}\equiv \left\{ X_{-j,t}, M_{t-1}, B_{j,t-1} \right\}$ shows the information set which includes the following variables: $X_{-j,t} = \left\{ X_{1,t}, X_{2,t}, . . ., X_{N,t} \right\}$ is a vector of explanatory variables, which includes the log returns of all the sample except for firm *j*. *N* is the number of firms, while $B_{j,t-1}$ is a vector, which includes the firms-specific characteristics (balance sheet variables). $\hat{\beta }_{j|\tilde{R}_j}$ is defined as follows $\beta _{j|\tilde{R}_j} = \left\{ \beta _{j|-j}, \beta _{j|M}, \beta _{j|B_j} \right\} ^ \top$. $\hat{D}_{j|\tilde{R}_j}$ captures the marginal effects of explanatory variables, where $\hat{D}_{j|\tilde{R}_j} = \left\{ \hat{D}_{j|-j}, \hat{D}_{j|M}, \hat{D}_{j|B_j} \right\}$. $\hat{D}_{j|-j}$ measures the spillover effect from all network to firm *j*. $g(\cdot )$ denotes the shape of the link function.

Finally, the tail-risk contagion is put in an $N \times N$ adjacency matrix (total connect matrix), with a set of nodes ($V=\left\{ 1, 2, ..., N \right\}$) and edges (*E*).

8 $$\begin{aligned} A= \begin{bmatrix} 0 &{} |\hat{D}^w_{1|2}| &{} |\hat{D}^w_{1|3}| &{} \cdots &{} |\hat{D}^w_{1|N}| \\ |\hat{D}^w_{2|1}| &{} 0 &{}|\hat{D}^w_{2|3}|&{} \cdots &{} |\hat{D}^w_{2|N}|\\ |\hat{D}^w_{3|1}| &{} |\hat{D}^w_{3|2}| &{} 0 &{}\cdots &{} |\hat{D}^w_{3|N}|\\ \vdots &{} \vdots &{} \vdots &{} \ddots &{} \vdots \\ |\hat{D}^w_{N|1}| &{} |\hat{D}^w_{N|2}| &{} |\hat{D}^w_{N|3}| &{}\cdots &{} 0 \end{bmatrix} \end{aligned}$$

The off-diagonal elements of the *i*-th row measures the impact from firm *i* to *j*, and the off-diagonal elements of the *j*-th column captures the level of risk tail spillover from firm *j* to *i*. *w* is the the estimation window. By definition, $\hat{D}_{j|i}$ is equal to zero when $j = i$. The rows represent the incoming edges, while the columns correspond to outgoing edges.

###  Connectedness measures

Following Härdle et al. (2016) and Wang et al. (2018), we used several connectedness measures. First, we employed the total connectedness (TC) index, which measures the total level of tail-risk spillovers. It is defined as follows:

9 $$\begin{aligned} TC=\sum _{j=1}^{N}\sum _{i=1}^{N} |\hat{D}_{j|i}^W|. \end{aligned}$$

Second, we computed two measures of sector connectivity: the incoming ($GC_{g,w}^{IN}$) and outgoing ($GC_{g,w}^{OUT}$) connectedness. $GC_{g,w}^{IN}$ is defined by the sum of incoming edges:

10 $$\begin{aligned} GC_{g,w}^{IN}= \sum _{j \in g} \sum _{i=1}^{N} |\hat{D}^w_{j|i}|. \end{aligned}$$

while $GC_{g,w}^{OUT}$ is the sum of outgoing edges:

11 $$\begin{aligned} GC_{g,w}^{OUT}=\sum _{i=1}^{N} \sum _{j \in g} |\hat{D}^w_{i|j}| \end{aligned}$$

where $g = 1, 2, 3, 4$ refers to the four sectors (wind, solar, energy efficiency and oil).

Finally, we calculated the relative influence (RI) index, defined as the ratio between the difference and the sum of out-tail interconnectedness and in-tail interconnectedness. Following Kenett et al. (2010), we defined the RI as follows:

12 $$\begin{aligned} RI_{sector}(m) = \frac{\hat{D}_{out}^W(m)-\hat{D}_{in}^W(m)}{\hat{D}_{out}^W(m)+\hat{D}_{in}^W(m)} \end{aligned}$$

Therefore, RI $\in [-1:1]$. A positive value indicates that the sector emits more risk than it receives (and vice-versa).

## Data description

To investigate the tail-risk interdependence network between oil and clean energy sectors, we collected the weekly closing prices of 32 listed firms during the period from 3 January 2011 to 25 October 2021.^2^ We chose the top eight firms in each sector analysed (oil, wind, solar and energy efficiency), according to their size (total asset) ranking. As a proxy for the clean energy market, we used the NASDAQ OMX Green Economy Index Family. In particular, we selected the eight top companies for each clean energy index: (i) NASDAQ OMX Wind Index (tracks companies that produce energy through wind power) and (ii) NASDAQ OMX Solar Index (tracks companies that produce energy through solar power). Furthermore, we included the eight top firms belonging to the MSCI Global Energy Efficiency Index (a market capitalisation-weighted index designed to maximise exposure to clean technology environmental themes^3^). It includes firms operating in products and services from alternative energy, sustainable water, green building, pollution prevention and energy efficiency. There are several papers that use these indices to illustrate the clean energy market (see Reboredo, 2015; Reboredo et al., 2017; Pham, 2019). Meanwhile, for the oil stock market, we selected the top eight firms of the NYSE Arca Oil Index.^4^ This is a price-weighted index of the world’s top oil firms that deal in the exploration, production and development of petroleum. To homogenise the sample, we selected only the firms listed before 2011. Table 1 shows each firm’s information, including its ticker code, full name, abbreviation, total assets and market capitalisation (December 2020). As we can see, oil firms have the largest size (total assets) and MCs.

We computed the weekly return of firm *i* on day *t* as $r_{i,t}=\ln \frac{P_{i,t}}{P_{i,t-1}}$ where $P_{i,t}$ is the closing price of firm *i* at week *t*. Following Härdle et al. (2016) and Wang et al. (2018), we included balance sheet variables, which incorporate the following: total asset/total equity ratio to capture leverage; market-to-book as a measure of stock price performance; and (total asset), taking firm size in terms of book value. Cubic spline interpolation (like Härdle et al., 2016) was applied to transform the quarterly variables into weekly data. Furthermore, following Adrian and Brunnermeier (2016), we collected some macro state variables which are used to capture the characteristics of the global economy. We included (i) the implied volatility index (VIX) reported by the Chicago Board Options Exchange, (ii) the ICE BofAML Option-Adjusted Spreads reported by the Chicago Board Options Exchange, (iii) the weekly MSCI Global index returns and (iv) the weekly Dow Jones Global Real Estate index returns. For each stock return, balance sheet data and macro state variables, there were 470 weekly observations. We obtained all data from the Datastream database.

**Table 1:** Company information

Ticker	Firm	Abbr.	Total Asset (2020) bn USD	MC (2020) bn USD	Rank of T.A.	Rank of MC
	*Panel A: oil*					
RDS.A	Royal Dutch Shell Plc	RDS	389.26	71.43	1	5
PTR	Petrochina Company Limited	PTR	380.74	6.53	2	21
XOM	Exxon Mobil Corp	XOM	332.75	174.28	3	2
BP	Bp Plc	BP	267.65	69.58	4	6
TOT	Total S.A.	TOT	266.13	114.92	5	4
CVX	Chevron Corporation	CVX	239.79	157.69	7	3
SHI	China Petroleum & Chemical Corporation	SHI	265.31	58.92	6	7
PBR	Petróleo Brasileiro S.A	PBR	190.03	30.55	8	14
	*Panel B: wind*					
SGRE.MC	Siemens Gamesa Renewable Energy, S.A.	SGRE	19.21	18.41	19	17
VWS.CO	Vestas Wind Systems A/S	VWS	22.23	46.79	17	9
2208.HK	Xinjiang Goldwind Science & Technology Co. Ltd.	GOLD	16.71	7.39	20	20
0658.HK	China High Speed Transmission Equipment Group Co Ltd	CHST	3.95	1.61	25	28
NDX1.DE	Nordex Se	NRDX	5.44	3.21	24	24
0182.HK	Concord New Energy Group Limited	CNE	2.98	0.51	27	31
SUZLON.NS	Suzlon Energy Limited	SE	0.89	0.57	31	30
IFN.AX	Infigen Energy	IFN	0.89	0.46	32	32
	*Panel C: solar*					
AMAT	Applied Materials Inc	AMAT	22.35	54.09	16	8
FSLR	First Solar, Inc.	FSLR	7.11	10.40	22	19
JKS	Jinkosolar Holding Co., Ltd.	JKS	8.14	1.83	21	27
CSIQ	Canadian Solar Inc.	CSIQ	6.53	3.06	23	25
OERL	Oc Oerlikon Corporation Ag	OERL	3.79	3.53	26	23
SPWR	Sunpower Corporation	SPWR	1.64	4.36	29	22
3576.TW	United Renewable Energy Co., Ltd.	URE	1.13	1.33	30	29
ATA.TO	Ats Automation Tooling Systems Inc.	ATA	1.74	1.94	28	26
	*Panel D: energy efficiency*					
9022.T	Central Japan Railway Company	CJRC	86.72	30.72	9	13
9020.T	East Japan Railway Company	EJRC	80.55	26.76	10	15
0066.HK	MTR Corporation limited	MTR	37.47	34.55	12	12
TSLA	Tesla, Inc.	TSLA	52.14	668.90	11	1
DLR	Digital Realty Trust, Inc.	DLR	36.07	39.08	13	11
006400.KS	Samsung SDI Co., Ltd.	SMG	19.97	39.69	18	10
ALSMY	Alstom S.A.	ALS	33.49	18.61	14	16
VMW	Vmware, Inc.	VMW	29.02	15.51	15	18

Listed oil and clean energy firms with tickers, abbreviation (Abbr.) total asset (T.A.), and market capitalisation (MC). They are classified by four types: (i) Oil firms, (ii) Wind firms, (iii) Solar firms and iv) Energy Efficiency firms. Total assets and market capitalisations are in billions of Dollar (USD) as of December 2020

In Table 2, we report the descriptive statistics for each energy firm during the sample period. We can observe a positive average stock return for 63% of the sample, indicating the considerable growth of these sectors in recent years. According to MCs, oil stock returns have the smallest values of standard deviation, suggesting that this market is less volatile (more stable) than the clean energy market. Skewness is most negative for oil stocks. This suggests a higher probability of declines in returns for the oil markets. The kurtosis statistic is greater than zero for all firms, i.e., implying a leptokurtic tail (fat tail), in agreement with the Jarque-Bera test that rejects the normality hypothesis of the unconditional distribution. All the returns series are stationarily verified using the augmented Dickey-Fuller (ADF) test, the ADF-GLS test and KPSS.

**Table 2:** Summary statistic returns

Firm	Mean	Minimum	Maximum	SD	Skewness	Kurtosis	J-B	ARCH-LM (10)	ARCH-LM (20)	ADF	ADF-GLS	KPSS
*Panel A: oil*												
RDS	$$-$$ 0.001	$$-$$ 0.307	0.272	0.038	$$-$$ 0.928	16.812	6735$$^{***}$$	282.05$$^{***}$$	279.24$$^{***}$$	$$-$$ 7.48$$^{***}$$	$$-$$ 7.56$$^{***}$$	0.05
PTR	$$-$$ 0.001	$$-$$ 0.149	0.143	0.039	$$-$$ 0.183	1.211	37.735$$^{***}$$	35.64$$^{***}$$	50.54$$^{***}$$	$$-$$ 8.43$$^{***}$$	$$-$$ 5.95$$^{***}$$	0.07
XOM	$$-$$ 0.001	$$-$$ 0.249	0.442	0.064	0.263	4.463	475.64$$^{***}$$	15.41$$^{*}$$	19.11	$$-$$ 8.21$$^{***}$$	$$-$$ 9.04$$^{***}$$	0.21
BP	$$-$$ 0.001	$$-$$ 0.312	0.233	0.039	$$-$$ 1.068	14.311	4929.2$$^{***}$$	249.01$$^{***}$$	247.69$$^{***}$$	$$-$$ 7.77$$^{***}$$	$$-$$ 5.03$$^{***}$$	0.03
TOT	0.001	$$-$$ 0.271	0.276	0.038	$$-$$ 0.447	12.733	3835.6$$^{***}$$	255.17$$^{***}$$	253.6$$^{***}$$	$$-$$ 8.05$$^{***}$$	$$-$$ 4.97$$^{***}$$	0.04
CVX	$$-$$ 0.001	$$-$$ 0.268	0.260	0.051	$$-$$ 0.283	3.704	330.57$$^{***}$$	48.89$$^{***}$$	65.61$$^{***}$$	$$-$$ 7.42$$^{***}$$	$$-$$ 7.66$$^{***}$$	0.11
SHI	$$-$$ 0.001	$$-$$ 0.141	0.158	0.028	$$-$$ 0.201	3.299	260.28$$^{***}$$	25.88$$^{***}$$	32.42$$^{**}$$	$$-$$ 8.48$$^{***}$$	$$-$$ 7.64$$^{***}$$	0.14
PBR	$$-$$ 0.001	$$-$$ 0.551	0.192	0.044	$$-$$ 4.061	46.944	53439$$^{***}$$	49.23$$^{***}$$	49.21$$^{***}$$	$$-$$ 7.44$$^{***}$$	$$-$$ 9.82$$^{***}$$	0.05
*Panel B: wind*												
SGRE	0.002	$$-$$ 0.202	0.211	0.057	$$-$$ 0.269	0.843	23.568$$^{***}$$	17.29$$^{**}$$	31.32$$^{***}$$	$$-$$ 6.61$$^{***}$$	$$-$$ 5.25$$^{***}$$	0.13
VWS	0.003	$$-$$ 0.3218	0.225	0.063	$$-$$ 0.587	2.835	222.27$$^{***}$$	43.87$$^{***}$$	92.85$$^{***}$$	$$-$$ 6.62$$^{***}$$	$$-$$ 10.01$$^{***}$$	0.24
GOLD	0.001	$$-$$ 0.251	0.253	0.065	0.210	1.11	32.753$$^{***}$$	10.87	28.29$$^{**}$$	$$-$$ 6.91$$^{***}$$	$$-$$ 8.51$$^{***}$$	0.24
CHST	$$-$$ 0.001	$$-$$ 0.242	0.298	0.068	0.251	1.319	46.91$$^{***}$$	29.13$$^{***}$$	42.11$$^{***}$$	$$-$$ 7.78$$^{***}$$	$$-$$ 9.39$$^{***}$$	0.15
NRDX	0.001	$$-$$ 0.379	0.242	0.069	$$-$$ 0.521	2.691	196.1$$^{***}$$	6.83	12.99	$$-$$ 7.38$$^{***}$$	$$-$$ 10.17$$^{***}$$	0.10
CNE	0.001	$$-$$ 0.314	0.223	0.057	0.191	2.953	208.83$$^{***}$$	30.48$$^{***}$$	34.67$$^{**}$$	$$-$$ 6.69$$^{***}$$	$$-$$ 9.18$$^{***}$$	0.30
SE	$$-$$ 0.003	$$-$$ 0.372	0.511	0.0891	0.941	5.791	872.89$$^{***}$$	32.47$$^{***}$$	43$$-$$ 24$$^{***}$$	$$-$$ 7.53$$^{***}$$	$$-$$ 8.91$$^{***}$$	0.10
IFN	0.001	$$-$$ 0.233	0.317	0.058	0.545	3.985	401.36$$^{***}$$	34.021$$^{***}$$	43.35$$^{***}$$	$$-$$ 8.11$$^{***}$$	$$-$$ 10.44$$^{***}$$	0.18
*Panel C: solar*												
AMAT	0.004	$$-$$ 0.236	0.144	0.043	$$-$$ 0.389	1.871	96.63$$^{***}$$	30.65$$^{***}$$	44.35$$^{***}$$	$$-$$ 7.89$$^{***}$$	$$-$$ 11.29$$^{***}$$	0.21
FSLR	$$-$$ 0.001	$$-$$ 0.358	0.339	0.072	$$-$$ 0.191	2.909	202.67$$^{***}$$	29.63$$^{***}$$	35.89$$^{***}$$	$$-$$ 7.11$$^{***}$$	$$-$$ 8.17$$^{***}$$	0.25
JKS	0.001	$$-$$ 0.411	0.377	0.102	0.014	1.168	32.161$$^{***}$$	56.37$$^{***}$$	59.46$$^{***}$$	$$-$$ 7.76$$^{***}$$	$$-$$ 5.47$$^{***}$$	0.11
CSIQ	0.002	$$-$$ 0.352	0.288	0.0837	$$-$$ 0.266	1.262	44.192$$^{***}$$	8.81	15.56	$$-$$ 7.23$$^{***}$$	$$-$$ 6.97$$^{***}$$	0.10
OERL	0.001	$$-$$ 0.235	0.213	0.043	$$-$$ 0.458	4.323	459.71$$^{***}$$	21.37$$^{**}$$	54.15$$^{***}$$	$$-$$ 7.83$$^{***}$$	$$-$$ 6.11$$^{***}$$	0.21
SPWR	0.002	$$-$$ 0.397	0.463	0.092	0.366	2.812	198.9$$^{***}$$	18.13$$^{**}$$	27.69$$^{*}$$	$$-$$ 6.86$$^{***}$$	$$-$$ 8.44$$^{***}$$	0.18
URE	$$-$$ 0.002	$$-$$ 0.261	0.325	0.061	0.145	2.861	194.59$$^{***}$$	35.04$$^{***}$$	52.35$$^{***}$$	$$-$$ 7.92$$^{***}$$	$$-$$ 10.95$$^{***}$$	0.15
ATA	0.003	$$-$$ 0.195	0.136	0.042	$$-$$ 0.2843	2.217	123.34$$^{***}$$	11.36	25.28	$$-$$ 8.11$$^{***}$$	$$-$$ 7.62$$^{***}$$	0.08
*Panel D: energy efficiency*												
CJRC	0.001	$$-$$ 0.135	0.123	0.033	$$-$$ 0.071	1.557	57.58$$^{***}$$	33.17$$^{***}$$	60.38$$^{***}$$	$$-$$ 8.44$$^{***}$$	$$-$$ 10.81$$^{***}$$	0.27
EJRC	0.001	$$-$$ 0.176	0.109	0.029	$$-$$ 0.556	3.571	329.19$$^{***}$$	7.96	36.59$$^{***}$$	$$-$$ 8.94$$^{***}$$	$$-$$ 10.15$$^{***}$$	0.23
MTR	0.001	$$-$$ 0.079	0.063	0.021	$$-$$ 0.555	1.279	67.353$$^{***}$$	6.86	25.15	$$-$$ 7.58$$^{***}$$	$$-$$ 7.39$$^{***}$$	0.05
TSLA	0.009	$$-$$ 0.358	0.341	0.071	0.057	2.652	166$$^{***}$$	52.06$$^{***}$$	59.37$$^{***}$$	$$-$$ 7.38$$^{***}$$	$$-$$ 7.41$$^{***}$$	0.21
DLR	0.002	$$-$$ 0.177	0.111	0.031	$$-$$ 0.653	1.925	127.47$$^{***}$$	10.57	27.39	$$-$$ 8.45$$^{***}$$	$$-$$ 10.43$$^{***}$$	0.05
SMG	0.002	$$-$$ 0.268	0.152	0.049	$$-$$ 0.256	2.204	120.64$$^{***}$$	13.79	25.98	$$-$$ 8.68$$^{***}$$	$$-$$ 7.02$$^{***}$$	0.37
ALS	$$-$$ 0.001	$$-$$ 0.237	0.225	0.044	$$-$$ 0.256	4.501	483.05$$^{***}$$	35.34$$^{***}$$	60.69$$^{***}$$	$$-$$ 7.95$$^{***}$$	$$-$$ 8.23$$^{***}$$	0.07
VMW	0.001	$$-$$ 0.228	0.158	0.045	$$-$$ 0.665	3.135	273.17$$^{***}$$	23.51$$^{***}$$	28.78$$^{**}$$	$$-$$ 7.94$$^{***}$$	$$-$$ 6.34$$^{***}$$	0.08

Descriptive statistics for weekly stock returns. The augmented Dickey-Fuller (ADF) statistic tests the null hypothesis of unit root, as well as the ADF-GLS test and KPSS, respectively. Each ADF and ADF-GLS statistic is negative and less than the test critical value at the 1% significant level, rejecting the null hypothesis of a unit root. The KPSS test shows that the null hypothesis is accepted, i.e, the stock returns are stationary

$${^{*}}, {^{**}}, {^{***}}$$ indicate 10%, 5%, 1% significance level

## Empirical findings

### Aggregate analysis

The VaR and CoVaR were estimated using a rolling window size of $n = 48$, corresponding to one year’s weekly data, and then $T = 520$ observation, with 38 independent variables. For example, when RDS is the dependent variable, the independent variables include 3 RDS company characteristics, 31 other firms’ returns and 4 economic state variables. Following Reboredo (2015) and Härdle et al. (2016), we set the quantile level at $\tau$ = 0.05.

**Fig. 1:**
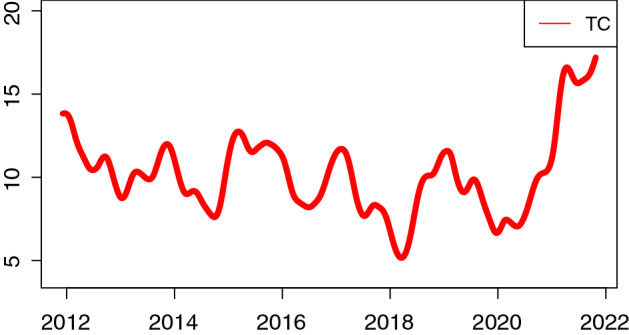
Total connection. *Notes:* Total connectedness of 32 firms from 21-11-2011 to 25-10-2021, $\tau$ = 0.05, window size *n* = 48, *T* = 520

In Fig. 1, we present the total connectedness (TC) index from December 2011 to October 2021. The figure shows that the total connection changes over time, highlighting some interesting patterns. In particular, we can observe how the initial high connection is interrupted by a short-lived decrease during 2012 with consequent three peaks with high interconnection values and a drastic reduction in 2018 (lowest point) and at the end of 2019. During 2020, the impact of COVID-19 was clear, making the connections increase considerably (higher peak). These spikes are highly correlated with various exogenous shocks or events (Managi et al., 2022).

**Fig. 2:**
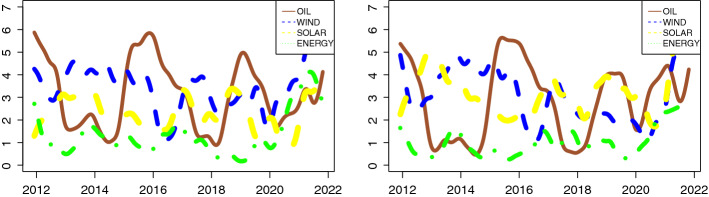
Incoming and Outgoing links. *Notes:* Incoming links (left-side) and Outgoing links (right-side) for four energy sectors, from 21-11-2011 to 25-10-2021. Oil: solid brown line, Wind: dashed blue line, Solar: dashed yellow line, Energy Efficiency: dash-dot green line. $\tau$ = 0.05, window size *n* = 48, *T* = 520

To better understand its dynamics, we plot in Fig. 2 the evolution of connectedness by sector, i.e., the contribution of each sector to the total dynamics. Recall that the TC is equal to the sum of incoming links and outgoing links for all four sectors. Hence, $TC = \sum _{m=1}^{4} GC^{IN}_m = \sum _{m=1}^{4} GC^{OUT}_m$. On the left-side, we can see the trend of incoming-links ($GC^{IN}$), while on the right-side the outgoing-links ($GC^{OUT}$). The patterns for both connections are almost identical except for the last period (COVID-19 era). Focusing on the dynamics of the oil sector (solid brown line), we can observe a high level of the link (in and out) in 2012, at the end of 2014, at the end of 2017, in mid-2019 and finally during the COVID-19 outbreak (March 2020). It is interesting to note that the $GC^{IN}$ ($GC^{OUT}$) trend is opposite to oil price (see Fig. 8 in “Appendix”). This finding corroborates the analysis of Xia et al. (2019), who found the same empirical evidence. This dynamic reflects five main events that occurred in the oil market: (1) the political upheaval in the Middle East and North Africa and the war in Libya in 2012, (2) the drop in oil prices from 2014 to 2016, (3) the China-United States trade war, (4) the reintroduction of sanctions on Iran in 2018-2019 and (5) the coronavirus crisis. Therefore, the results suggest that the uncertainty about oil price shock is one of the key factors in the risk dynamics for oil companies. In fact, many studies (Kilian & Park, 2009; Gupta, 2016; Kilian & Vigfusson, 2017; Lv et al., 2020) highlighted the positive relationship between oil price and oil firm performance. A rise in oil prices increases the performance of the sector; hence, there is a reduction in risk. The contrary effect when the oil price is low. In this case, with the same factors of production, the profit for oil companies is more moderate; this translates into increased risk. Meanwhile, we can see the opposite dynamic for the clean sector. For this market (wind, solar and energy), we can see an increase in interconnection from 2013 to 2015, a reduction in 2016 and again an increase between 2017 and 2021. This evolution is in line with the important events related to climate change. For example, we can note the positive impact, i.e., interconnection reduction, of the Paris Agreement (December 2015) or even COP-24 at the end of 2018. A possible explanation for the countercyclical trend between these markets can be found in the inherent link between uncertainty (volatility) and return. According to the substitution effect theory, high oil prices lead companies to use alternative energies. This implies an increase in returns of clean energy firms (Kumar et al., 2012; Baldi et al., 2014). In contrast, a decrease in oil prices reduces the use of renewable energy because of the high costs associated with the construction and installation of these energy systems (Uddin et al., 2019). However, the clean energy dynamics connectedness seems unaffected by uncertainty in the oil market. Indeed, as pointed out by Ferrer et al. (2018), the relationship between these markets has changed over time. For example, this decoupling of the renewable energy sector from the oil market depends on the fact that these two sectors ‘no longer compete in the same markets’ (Ferrer et al., 2018).

The graphical analysis of the interconnections (incoming and outgoing) shows that the TC trends can be traced by the incoming or outgoing connection of the four sectors. The TC during the years 2013 and 2015 was caused by the incoming and outgoing connection of the wind and solar sectors. The link of the oil industry generates TC in the second circle. The TC in the period 2017 was caused one more time by the connection between wind and solar. Finally, the TC in the last peak is reflected by all sectors because of the COVID-19 outbreak. It is interesting to note the marginal effect of the energy sector. Its connections, both incoming and outgoing, are much lower than those of the other industries and are quite constant over time.^5^

**Fig. 3:**
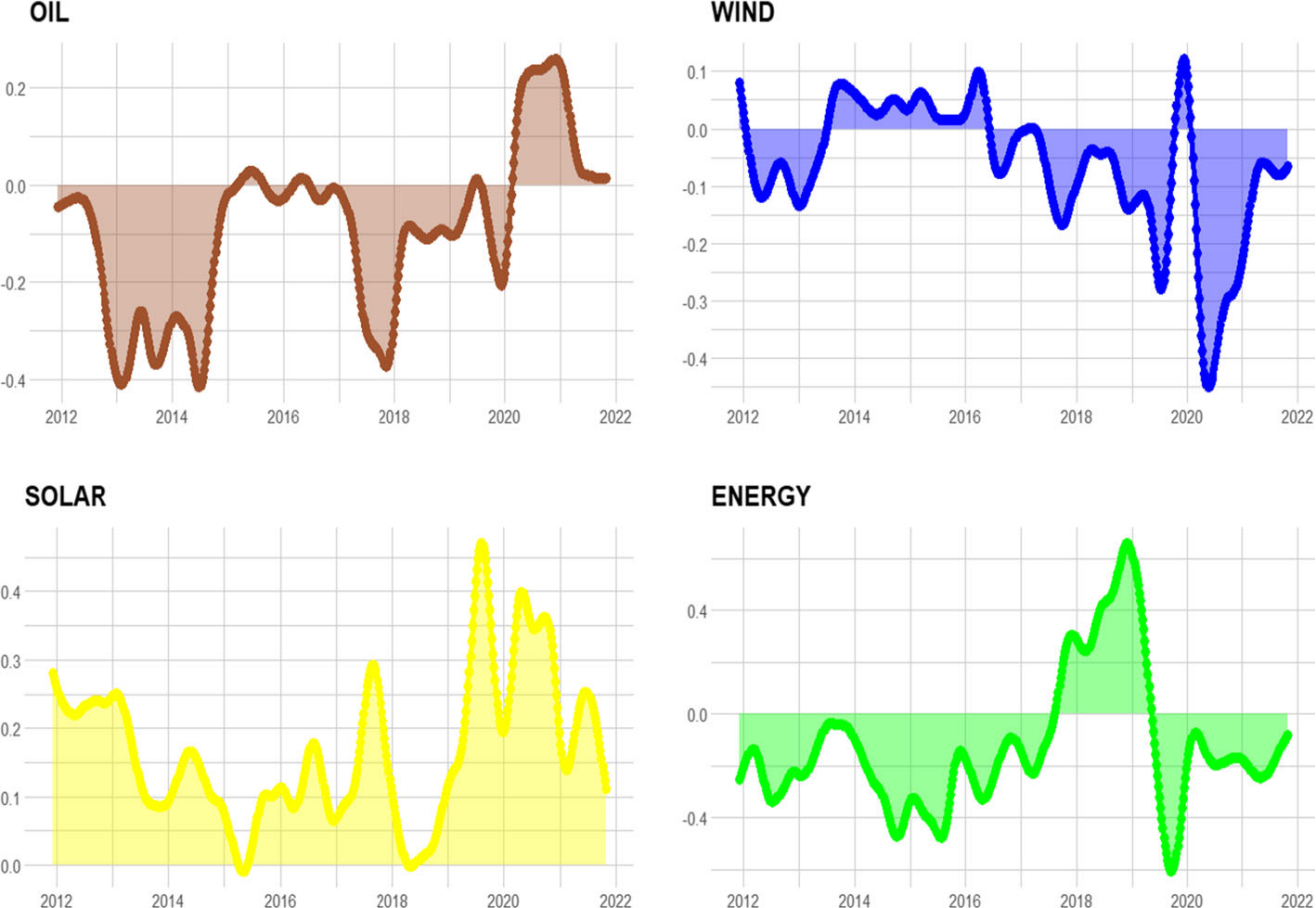
Relative Influence. *Notes:* Dynamic relative influence of each energy sectors, from 21-11-2011 to 25-10-2021. Oil: brown, Wind: blue, Solar: yellow, Energy Efficiency: green. $\tau$ = 0.05, window size *n* = 48, *T* = 520

To analyse the directional information, in Fig. 3 we show the relative index (RI). Focusing on RI, it is possible to notice whether a sector is a net transmitter or a net receiver of tail-risk spillover effects. A positive value in the index means that the sector in question is a net tail-risk transmitter to all others, while a negative value indicates that this sector is a net tail-risk receiver. The oil sector is a net tail-risk receiver in the pre-COVID-19 period. The value of tail risk transmitted from the clean sector to the oil sector was higher than the degree of tail risk carried in the opposite direction. These results are consistent with the studies of Pham (2019) and Ferrer et al. (2018), which investigated the connection between the aggregate clean energy stock market and the oil price. Both works, using Diebold and Yılmaz (2012, 2014)’ models, showed that the value of shock transmitted from clean energy stocks to oil price was higher than the amount of shock transmitted in the opposite way. However, during the coronavirus outbreak, the RI documents how the oil sector changes its role from net tail receiver to net tail emitter. As we can note, the oil sector plays a key role in risk spread for the energy sector. Meanwhile, we can observe how the solar sector is always a net transmitter of tail risk, confirming the analysis of Foglia and Angelini (2020), Pham (2019) and Reboredo (2015). In particular, Reboredo (2015) showed how the contribution to systemic risk for the solar energy sector has a more significant impact on the upside (81% on average) than the downside (58% on average) of extreme fluctuations in oil prices. Of interest is the dynamics for the energy sector, which during 2018 issued tail risk. Indeed, the global stock fell by 7.1% in 2018, according to the MSCI World Index. This decrease generated a global increase in risk as the TC shows. The sectors with the worst performance were resources and basic materials (which are used in construction) as well as the automotive industry. Besides, according to the report of IEA (2019a), energy intensity improvement continued to slow in 2018 with its slowest rate since 2010. For example, in the transport sector, despite improvements in vehicle efficiency, energy intensity fell because of sales reductions.

**Fig. 4:**
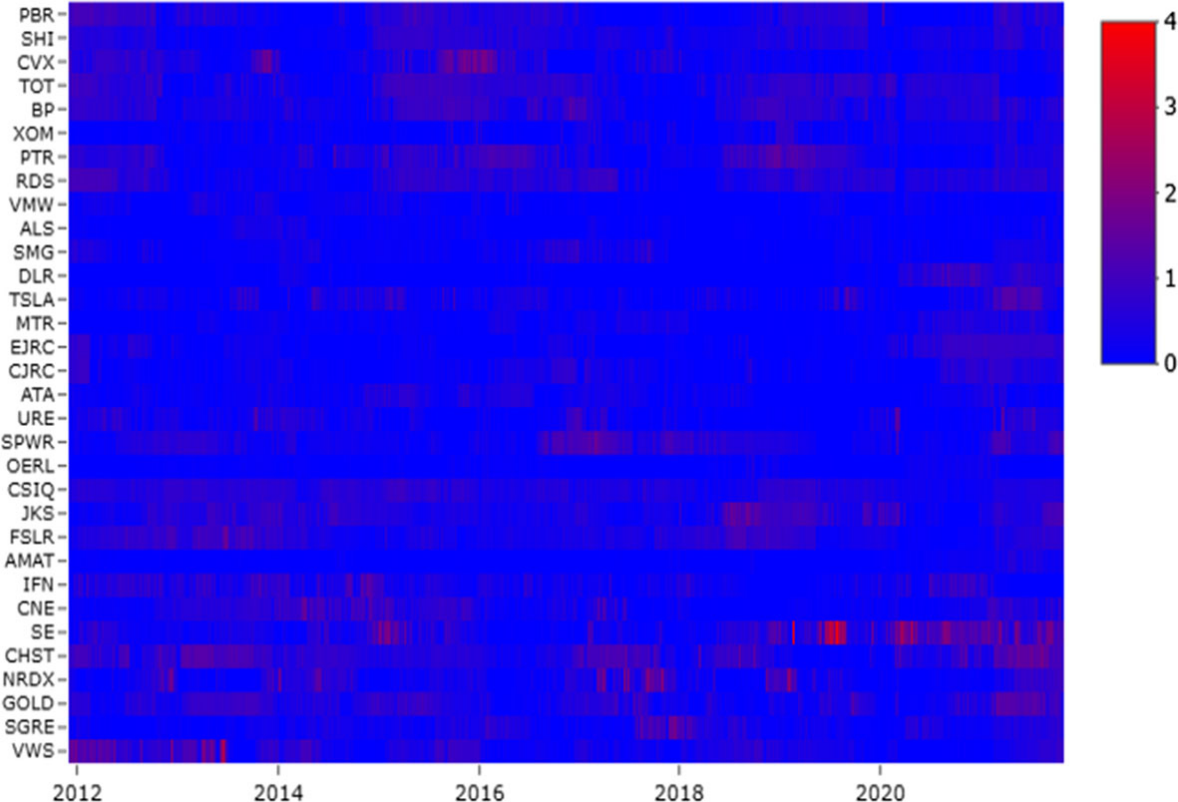
Firm Incoming Link ($F^{IN}$). *Notes:* $F^{IN}$ measures the level of incoming connectedness of firms. The full name of each firm is shown in Table 1

Our investigation of risk spillover revealed that there is heterogeneity in tail-risk transmission. This finding is quite informative for policymakers or investors regarding the evolution of the energy market. For example, knowing the sector that emits the most risk is useful for investors who want to incorporate the clean sector into their diversification strategies.

### Firm level analysis

**Fig. 5:**
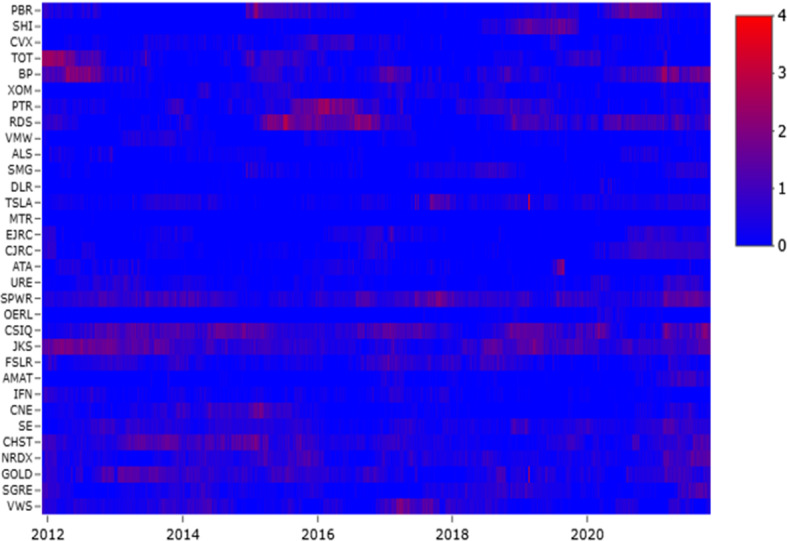
Firm Outgoing Link ($F^{OUT}$). *Notes:* $F^{OUT}$ measures the level of outgoing connectedness of firms. The full name of each firm is shown in Table 1

In this section, we analyse the tail risk spillover at the firm level. In particular we compute the firm incoming ($F^{IN}$) and outgoing ($F^{OUT}$) connectedness.^6^ Figures 4 and 5 plot the evolution of $F^{IN}$ and $F^{OUT}$ in dynamic TENET, for each firm in each sector, respectively. We can observe how the incoming and outgoing connections are time-varying following the TC pattern. Focusing on oil firms (up), we can see three main periods that have characterised the risk reception: 2012–2013, 2015–2016 and 2019–2020. Meanwhile, the wind and solar companies receive high levels of risk almost uniformly throughout the period (peaks in 2014 and 2018), as well as the energy sector, which receives less risk than all the others. Finally, at the start of 2020, the collapse of the financial and economic markets due to the COVID-19 pandemic affected both the brown and green sectors. Therefore, based on our sample, we could draw attention that COVID-19 could be a driver for systemic risk in clean energy and oil firms. From this starting point, we propose policy implication to control the adverse effects of COVID-19 regulations on commodity markets.

The distribution of $F^{OUT}$ (Fig. 5) is quite similar to that of $F^{IN}$. In the first period (2012 to mid-2013), two oil companies (TOT and BP) and one in the solar sector (JKS) had the highest $F^{OUT}$ values. From mid-2013 to mid-2014, the clean energy sector emitted the highest risk, with two solar firms (CSIQ and JKS) and four wind companies (SE, CHST, GOLD and VWS). These years have been turbulent for the clean energy market. Indeed, according to REN21 (2014)’ report, the economic crisis and political uncertainty have increased the capital cost. Therefore, countries have reduced financial support for clean energy. In the European context, many renewable companies have become bankrupt, and this has caused international risk spillover effects. During the drop-oil phase, oil companies emitted the highest tail risk, such as RDS, PTR, CSX, and PBR. This period was characterised by increased market uncertainty regarding future oil prices. This uncertainty was caused by plentiful oil supplies because of shale production and excess supplies by oil producers (Awartani et al., 2016). These firms, trying to maintain their market shares in a context of weakening demand (financial crisis and global economic easing), have contributed to the uncertainty in the energy markets, emitting more risk. During the past year of the analysis (2020), each sector issued tail risks, highlighting how uncertainty in the stock markets has also played an important role in the energy sector.

**Fig. 6:**
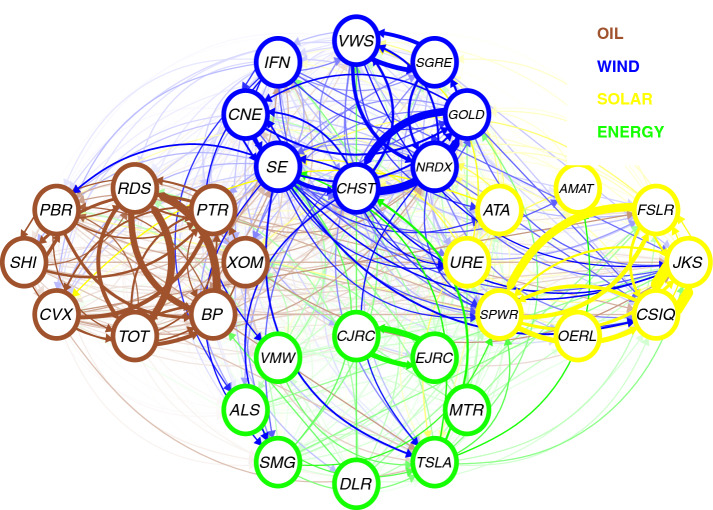
Spillover Network. *Notes:* A network representation of the weighted adjacency matrix (overtime). Oil: clockwise 8 firms from RDS to CVX (left brown), Wind: clockwise 8 firms from WWS to IFN (upper blue), Solar: clockwise 8 firms from AMAT to ATA (right yellow), Energy Efficiency: clockwise 8 firms from CJRC to VMW (lower green)

Figure 6 shows the spillover network of pairwise directional connectedness for the 32 energy firms during the whole period. The plot is an elliptical network representation of the weighted adjacency matrix computed using the TENET framework. Table 3 reports the top eight firms in term of edges, i.e. the directional connectedness^7^ from firm *i* to firm *j*.

**Fig. 7:**
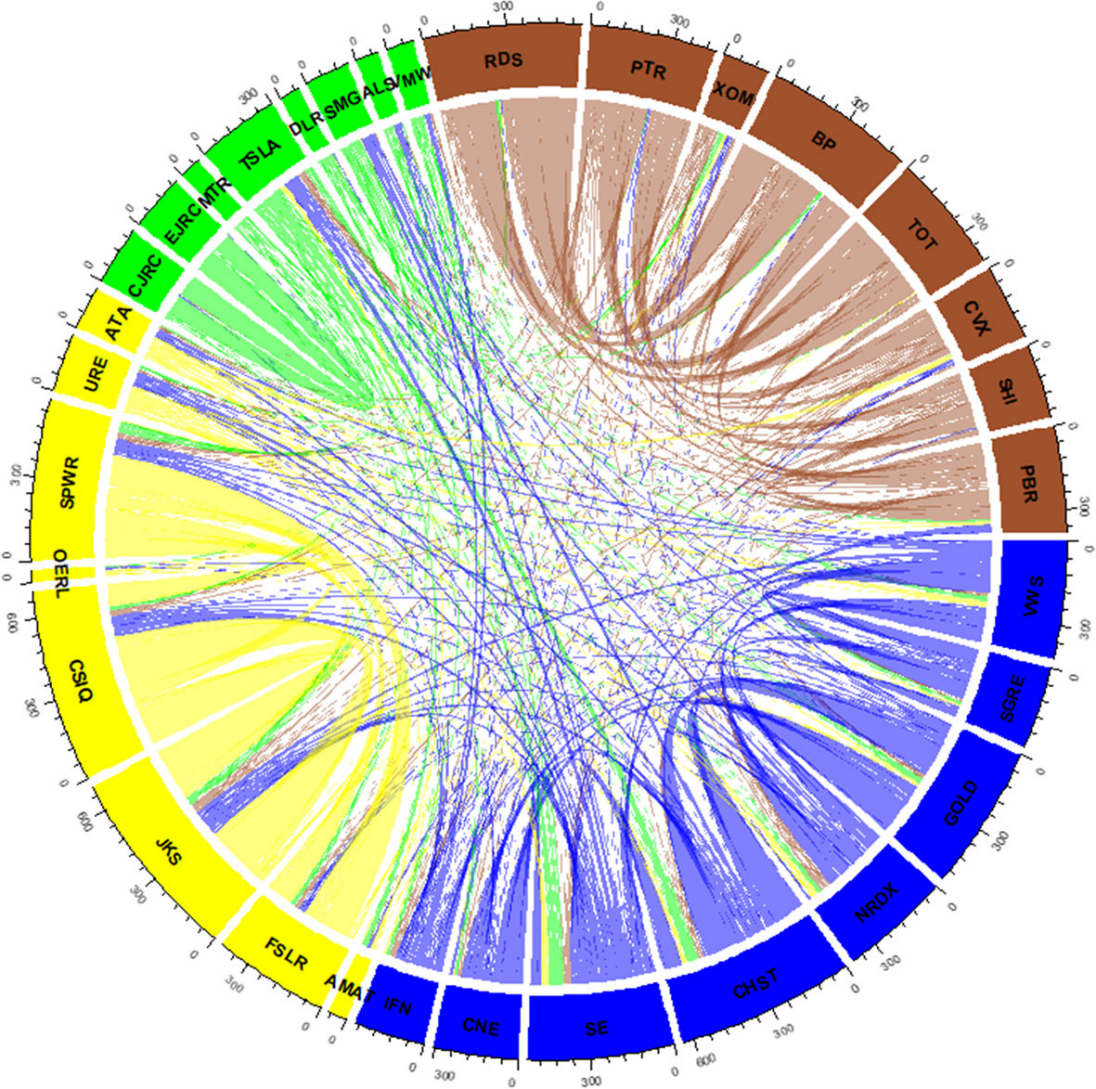
Spillover Chord. *Notes:* A chord plot representation of the weighted adjacency matrix (overtime). Oil: clockwise 8 firms from RDS to CVX (brown), Wind: clockwise 8 firms from WWS to IFN (blue), Solar: clockwise 8 firms from AMAT to ATA (yellow), Energy Efficiency: clockwise 8 firms from CJRC to VMW (green)

**Fig. 8:**
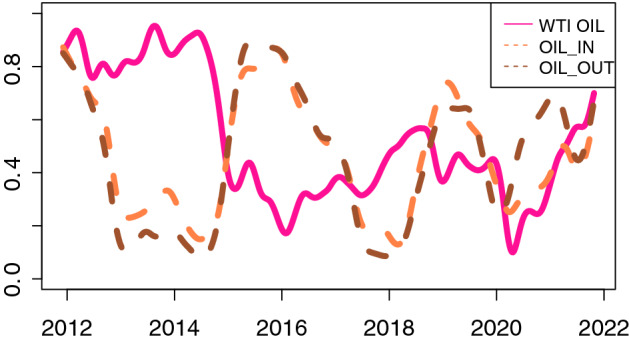
WTI oil price and OIL connectedness. *Notes:* WTI oil price (solid pink line) and OIL $GC^{IN}$ (dot light-brown line) and OIL $GC^{OUT}$ (dot brown line), from 21-11-2011 to 25-10-2021. Zero mean and unit variance standardise

Several findings arose from the network spillover. We found several strong connections between solar firms. In particular, CSIQ and JKS were the largest tail-risk emitters, followed by SPWR, which transmits tail risk to FSLR. In the fourth and fifth positions were companies in the wind sector (GOLD and CHST, respectively), which transmit risk to each other. It should be pointed out that GOLD and CHST are both Chinese wind firms. These companies can be considered similar in terms of portfolio diversification (similar market capitalisation). This factor may explain their tail-risk spillover relationship. Finally, the firms in the oil sector, namely RDS, BP, and TOT (as in Restrepo et al., 2018). These results suggest that most of the strong tail-risk spillovers are from solar to solar, from wind to wind, and from oil to oil. Hence, we can observe a bright sector cluster. Each firm receives (emits) more tail risk from (to) its sector. This implies that the tail risk of one sector is likely to spread to the same industry but not necessarily to others. In fact, we found (i) a rather low connection at a system level, (ii) a sector cluster and (iii) a high link within the sector. Figure 7 highlights these intrasectoral relationships, showing the almost absence of internal links. There are links between wind, solar, and energy and a few links between oil and clean energy. This result contradicts the common perception that the oil market plays a key role in the stock market dynamics of clean energy companies. According to Ferrer et al. (2018), the performance of these firms is probably more linked to factors such as technology innovation, legislation or capital spending than to the oil market. This disconnect between these markets also lies in the fact that they are no longer competitors (direct substitutes), and then they are used to satisfy different parts of the global energy demand. These results are in line with several works that found a poor relationship between oil price and the clean energy market (Henriques & Sadorsky, 2008; Sadorsky, 2012a; Ahmad, 2017; Ferrer et al., 2018; Pham, 2019). It is important to keep in mind that our analysis, unlike the research above, does not focus on oil prices but on oil firms. Meanwhile, we found that even at the corporate level, the two sectors are quite distinct. One of the possible recommendations is the hedging position from two types of commodities. Hence, our findings could shed new light on the diversification strategy for investors to obtain the optimal portfolio when investing in these markets.

**Table 3:** Top 8 spillover from firm *i* to *j*

Rank	From	To	Sum
1	CSIQ	JKS	183.68
2	JKS	CSIQ	164.11
3	SPWR	FSLR	132.73
4	GOLD	CHST	130.39
5	CHST	GOLD	126.58
6	RDS	BP	122.57
7	RDS	TOT	113.82
8	BP	RDS	99.86

Top 8 directional edges from firm *i* to *j*. Following Härdle et al. (2016), we compute the raking by summing the absolute value of the partial derivatives, $\tau$ = 0.05, window size *n* = 48, *T* = 520

To further analyse the connectedness of each firm, in Table 4, we report the top eight companies in terms of incoming and outgoing links. Among the top eight energy firms classified by the in-link, there are three solar (CSIQ, JKS and FSLR), three oil (RDS, TOT and BP) and two wind companies (CHST and SE). As we can see, most of these companies (except those in the oil sector) have a small or moderate market capitalisation. In the right part of the table, we report the top eight energy firms classified by the out-link. Likewise, almost all of them have a modest (small) market capitalisation, such as JKS, CSIQ and SPWR. Hence, we can see that solar companies are the ones with the highest risk, which shows how this sector has been extremely active in risk transmission in recent years (Kim et al., 2019; Foglia & Angelini, 2020). In fact, increasing oversupply and overcapacity in the solar sector have reduced the profitability of this industry (Bohl et al., 2013; Rezec & Scholtens, 2017). These poor performances imply high risks, which could be aggravated by the existence of regulatory risk (Wüstenhagen & Menichetti, 2012). This finding corroborates the analysis of Kazemilari et al. (2017). Using the minimum-spanning-trees approach, the authors found that solar firms (such as First Solar, Inc.) are the most important within the network, and these stocks play a significant role in renewable energy development.

**Table 4:** Top IN and OUT spillover firms

Rank	Firms	IN-Links	MC rank	Firms	OUT-Links	MC rank
1	CHST	326.81	28	CSIQ	448.48	25
2	SE	307.69	31	JKS	447.25	27
3	JKS	248.19	27	SPWR	373.73	22
4	CSIQ	246.66	25	RDS	224.81	5
5	TOT	244.99	4	CHST	312.71	28
6	BP	243.52	6	BP	296.02	6
7	GOLD	240.07	20	GOLD	280.06	20
8	RDS	234.71	5	PTR	222.66	21

Top 8 firms ranked by IN and OUT links, with market capitalisation ranking (MC)

Based on our analysis, we can see, on the one hand, how solar and wind emit much risk within the system. This is coherent with the results found by Lundgren et al. (2018), Sadorsky (2012b) and Henriques and Sadorsky (2008), who showed that investments in the clean sector are riskier than those in the nonrenewable sector. On the other hand, we found the marginal role of the energy sector. In fact, while solar, wind and oil companies dominate both incoming and outgoing connections, energy firms show a smaller contribution in terms of risk transmission. These results are fully consistent with those reported by Reboredo (2015), who found that the green technology sector has systemic risk values (CoVaR) lower than the wind, solar and oil price index. The less risky nature of this sector depends on the fact that the performance of these investments is linked to investments in other types of technology applications that offer less uncertainty. Moreover, it is a sector that generates profit opportunities that are less dependent on government stimuli and price oil dynamics. Overall, our findings can be helpful, both in portfolio investment strategies (considering systemic risk) and in designing regulatory policies. We estimate the total connection and identify the companies that transmit and receive tail risk in a way that would allow managers to mitigate the risks arising from the energy financial markets. The information can enable policymakers to comprehend the relationship between oil and clean energy markets.

## Concluding remarks

In this study, we analysed the interconnection (tail risk) between 32 companies in the clean energy and oil sectors from 3 January 2011 to 25 October 2021. For this purpose, we employed the TENET risk model. The analysis of the total and cross-sector interconnections of the green and brown energy sectors represent a significant contribution. Although several papers have studied the correlation between these two markets, none has empirically examined the spillover effects at the firm level.

Our results can be classified into three parts. First, we found the TC between the markets varies during the period analysed with a downward trend. The incoming and outgoing links dynamics of the oil sector are opposed to the behaviour of oil prices. Therefore, the uncertainty in oil price plays a critical role in the risk dynamics for oil companies. Focusing on the clean market, we can see how the patterns are opposite, showing how the two sectors are distinct. Second, the results showed that several small firms have a high link, highlighting how the risk is not just a matter between big firms. Third, the relationships between energy firms tended to be intrasectoral (sector cluster). That is, each sector receives (emits) risk from (to) itself. The empirical analysis showed a clear decoupling of the renewable energy sector from the oil market.

The study of interconnections is particularly valuable for policymakers, as it provides a clear framework to enable protection against contagion and promote energy market stability. For example, the TC can be used as a warning indicator for energy system distress. Moreover, the lack of a strong connection between oil and clean energy companies means that the alternative energy sector did not need specific policies to protect against the impact of the oil energy sector. Furthermore, the analysis of risk interconnections can help investors with their portfolio strategies. The study shows that investing in oil companies is less risky when oil prices are high. Also, by analysing the risk contribution for each firm (sector), we help investors in their investment strategies to include companies (sector) depending on their level of tail risk in that particular period.

The main limitation of our research is its limited dataset. In fact, the data used in our study did not include all listed companies in the oil and clean energy sector (only 32 firms) because to homogenise the sample, we eliminated firms for which we have limited data. The second limitation, stemming from the first, is that we only considered the largest firms. An analysis also focused on small businesses would certainly provide deeper insights into the interconnections of these markets. History shows that companies can be too big to fail as well as too interconnected to fail. Therefore, we could extend the analysis using new econometric techniques that need fewer time-series data or different frequencies (e.g., MIDAS model). It would also be interesting to study how oil price affects the two markets to have more clarity on the evolution of spillover risk at the firm level. For instance, a further extension could be to test Sadorsky (2012b)’ study, i.e., whether high oil prices imply a greater systemic risk for clean sector firms from a network-connectedness perspective.

## Appendix

See Fig. 8.

## Footnotes

1 IEA (2019b) expected an increase in global energy demand by 1.3% each year until 2040.

2 The choice for our clean energy and oil datasets was based on the satisfaction of two criteria. First, they must be the largest companies (total assets). This criterion ensures that the firms chosen are among the largest players in the market. Second, they must be listed before 2011, in order to have significant observation for the estimation of tail risk. Obviously, it was preferable to have a longer time series, but we have selected this period with the maximum availability of data for the different companies to minimise the missing data. For example, 15 companies belong to the NASDAQ OMX Wind Index. Considering these two criteria, we selected 8 wind firms. Therefore, to homogenise the sample, we have chosen the top eight firms for each sector. Further, we used weekly data because the software cannot allocate the amount of memory needed to estimate the TENET model with daily data. Moreover, we opted for the usage of weekly data to apply the TENET method because the modelling is more adequate with that type of data according to previous studies (Härdle et al., 2016).

3 See https://www.msci.com/documents/10199/7053382b-edcd-4d35-8bb0-44e798ba65f2.

4 We selected the index constituents in February 2020. For NASDAQ OMX Wind Index, NASDAQ OMX Solar Index and NYSE Arca Oil Index, we selected companies from Yahoo! Finance. At the same time, for the MSCI Global Energy Efficiency Index, we chose firms from the MSCI document at https://www.msci.com/documents/10199/7053382b-edcd-4d35-8bb0-44e798ba65f2.

5 The findings are still valid when we consider an alternative proxy for the energy efficiency market (proxied by the NASDAQ OMX Smart Grid Index) into our connectedness framework. The results are available upon request.

6 We define the firm incoming edges as: $F^{IN} = \sum _{i=1}^{N} |\hat{D}^w_{j|i}|$. While, the firm outgoing edges is defined as: $F^{OUT}=\sum _{i=1}^{N} |\hat{D}^w_{i|j}|$.

7 Directional connectedness is defined as follows: $DC_{j|i} = |\hat{D}_{j|i}|$.
